# Diagnostic Performance of the Triglyceride-Glucose Index in Screening for Gestational Diabetes Mellitus at 24–28 Weeks of Gestation

**DOI:** 10.3390/diagnostics15212682

**Published:** 2025-10-23

**Authors:** Saliha Sağnıç, Tuğba Gül Yılmaz, Addule Serhanoğlu Seçen, Mustafa Bağcı, Selin Güney, Mert Cenker Güney, Ayşegül Atalay

**Affiliations:** 1 Department of Gynecologic Oncology, Clinic of Obstetrics and Gynecology, Van Training and Research Hospital, Van 65300, Türkiye; 2 Clinic of Obstetrics and Gynecology, Van Training and Research Hospital, Van 65300, Türkiye; drtugbagulyilmaz@gmail.com (T.G.Y.); Adule.serhan@gmail.com (A.S.S.); selinyilmaz64@yahoo.com (S.G.); dr.mertcenkerguney.akd@gmail.com (M.C.G.); 3 Department of Perinatology, Clinic of Obstetrics and Gynecology, Van Training and Research Hospital, Van 65300, Türkiye; mustafabagci@outlook.com.tr (M.B.); draysegulatalay@gmail.com (A.A.)

**Keywords:** gestational diabetes mellitus, insulin resistance, oral glucose tolerance test, screening, triglyceride-glucose index

## Abstract

**Background/Objectives**: The objective of this study was to assess the diagnostic accuracy of the Triglyceride-Glucose (TyG) index for screening gestational diabetes mellitus (GDM) at 24–28 weeks of gestation, to determine its optimal diagnostic threshold, and to compare its predictive performance with conventional lipid ratios (LDL/HDL, TG/HDL, and TC/HDL). **Materials and Methods**: We retrospectively analyzed 440 pregnant women with singleton pregnancies who underwent a 75 g oral glucose tolerance test (OGTT) between January and July 2025. The TyG index and lipid ratios were calculated, and their associations with GDM were evaluated. Subgroup analyses were conducted to assess the efficacy of the TyG index in predicting GDM, using logistic regression to estimate odds ratios (ORs) with 95% confidence intervals (CIs), and receiver operating characteristic (ROC) curve analysis along with restricted cubic spline modeling to evaluate diagnostic performance and determine the optimal cutoff value. **Results**: The overall prevalence of GDM, as defined by the IADPSG (International Association of the Diabetes and Pregnancy Study Groups) criteria, was 22.7%. The median TyG index was significantly higher in the GDM group compared with the non-GDM group (9.1 vs. 8.9, *p* = 0.001). The TyG index was a significant predictor of GDM (*p* < 0.05), with each one-unit increase associated with significantly higher odds of GDM (OR = 12.29), after adjusting for covariates. ROC analysis demonstrated an AUC of 0.716 (95% CI: 0.627–0.793, *p* < 0.001) for the TyG index, and the optimal cut-off value was identified as 9.35, yielding a sensitivity of 38.5% and a specificity of 96.5% and a negative predictive value of 83.7%. Subgroup analyses indicated that the TyG index had limited discriminative ability for predicting GDM in both the post-load and insulin-requiring groups. Among conventional lipid ratios, TG/HDL demonstrated the highest predictive performance (AUC = 0.587), while LDL/HDL (AUC = 0.483) and TC/HDL (AUC = 0.509) demonstrated low predictive accuracy. Compared with conventional lipid ratios, the TyG index demonstrated superior predictive performance. **Conclusions**: A higher TyG index was positively associated with the development of GDM and showed better predictive ability than conventional lipid ratios. However, its low sensitivity limits its use as a standalone diagnostic tool, suggesting it may be most useful when combined with other clinical parameters.

## 1. Introduction

Gestational diabetes mellitus (GDM) is characterized by disruptions in glucose homeostasis, resulting in hyperglycemia during pregnancy. This metabolic disorder poses significant short- and long-term risks to both maternal and fetal health. Early detection and timely intervention in GDM are crucial to ensuring optimal health outcomes for both the mother and the offspring [1]. However, there is a notable lack of global consensus regarding the optimal strategy for GDM screening. A universal approach, endorsed by organizations such as the American Diabetes Association, is advised for all pregnant women during their second trimester [2]. In contrast, a number of European medical bodies favor a selective strategy based on individual patient risk profiles [3]. While universal screening undoubtedly improves detection rates, it concurrently escalates the clinical workload, the number of diagnostic procedures performed, and the overall degree of medical intervention. A key challenge to selective screening is the ongoing debate over which risk factors most accurately identify at-risk individuals, highlighting a critical area for further investigation.

The 75 g Oral Glucose Tolerance Test (OGTT) is a standard test used to diagnose GDM during pregnancy. While generally safe and well-tolerated, it can cause side effects like nausea and vomiting in a significant number of women. Since vomiting can invalidate the test, a repeat procedure is often necessary, creating a practical and financial burden. This underscores the need for more tolerable and accurate diagnostic alternatives.

In the screening of pregnant individuals for GDM, careful selection of the most practical and reliable diagnostic test is crucial, as the underlying pathophysiology of GDM is primarily characterized by impaired insulin secretion or reduced insulin sensitivity [4]; therefore, surrogate markers of insulin resistance may serve as effective predictors for its development. If such markers can be identified in pregnant women between 24 and 28 weeks of gestation, the number of required OGTTs may be significantly reduced.

Although several biochemical parameters have been proposed for the assessment of insulin resistance, the euglycemic-hyperinsulinemic clamp remains the gold standard owing to its high accuracy; however, its invasive nature and methodological complexity limit its feasibility in routine clinical practice [5]. The Homeostatic Model Assessment of Insulin Resistance (HOMA-IR) index represents the most established and commonly used surrogate marker for insulin resistance [6]. An elevated HOMA-IR has been shown to be a strong predictor of diabetes development. Moreover, higher HOMA-IR values have been independently associated with an increased risk of prediabetes [7,8]. In addition, conventional lipid ratios including LDL/HDL (Low-density lipoprotein/High-density lipoprotein), TG/HDL (Triglyceride/High-density lipoprotein), and TC/HDL (Total cholesterol/High-density lipoprotein), have been investigated as surrogate markers of insulin resistance and risk of GDM [9,10,11]; however, their diagnostic accuracy and clinical cut-offs remain inadequately defined [11,12,13]. In this context, the triglyceride-glucose (TyG) index, first introduced in 2008, has emerged as a validated surrogate marker, demonstrating comparable performance to both the clamp technique and HOMA-IR in detecting insulin resistance in apparently healthy populations [1,14]. The TyG index is a cost-effective and practical tool, requiring only two routine measurements: triglycerides and fasting plasma glucose.

The TyG index, an insulin-independent ratio, has emerged as a valuable marker for identifying insulin resistance and has been increasingly investigated as a predictive tool for GDM [15,16]. While recent evidence indicates that the TyG index may function as a dependable predictive marker for GDM when assessed at 24–28 weeks of gestation [17], findings across studies remain inconsistent, with notable heterogeneity in reported cut-off values, sensitivities, and specificities [18,19,20]. In fact, a study conducted in Latin American women reported no significant association between early pregnancy TyG and GDM [19]. Additionally, several studies have highlighted the limited diagnostic value of TyG index [21]. Such variability is likely multifactorial, arising from differences in the pathophysiological pathways of GDM, divergent study population characteristics, variations in sample sizes, and, crucially, the gestational timing of the assessment. The TyG index is most effective in the second trimester (24–28 weeks), because the marked rise in insulin resistance at this stage enhances its diagnostic accuracy and reduces the false negatives observed in early pregnancy. In parallel, conventional lipid ratios such as TG/HDL, LDL/HDL, and TC/HDL, although well-established markers of insulin resistance in cardiovascular disease, lack standardized clinical cutoffs in the context of GDM. Nonetheless, emerging evidence suggests that elevations in these ratios may also be associated with an increased risk of GDM diagnosis during the second trimester [10,11,13,22,23,24,25,26].

The existing heterogeneity in findings, particularly regarding the gestational timing of assessment and the lack of standardized cutoffs for both the TyG index and conventional lipid ratios, underscores the need for further research. Therefore, we conducted this study to evaluate the predictive value of the TyG index and lipid ratios for GDM in our study population.

## 2. Materials and Methods

This retrospective study was approved by the Institutional Ethics Committee of the University of Health Sciences Van Education & Research Hospital (approval no: GOKAEK/2025-06-04; date: 8 August 2025). The committee granted a waiver of informed consent due to the use of anonymized patient data from records between January and July 2025. The study was conducted in accordance with the ethical principles of the Declaration of Helsinki.

Following ethics approval, we retrospectively analyzed the electronic hospital records of all pregnant women who underwent universal screening for GDM via a 75 g OGTT at 24–28 weeks’ gestation as part of routine antenatal care at our tertiary referral center (Clinic of Obstetrics and Gynecology, Van Training and Research Hospital, Van, Türkiye). Women were excluded based on the following criteria: age < 18 or >40 years; pre-existing type 1 or 2 diabetes; current use of medications affecting glucose, lipid, or insulin metabolism; pre-existing metabolic, cardiac, endocrine, hepatic, renal, pancreatic, autoimmune, or malignant disorders affecting glucose, lipid metabolism or insulin physiology; substance use (alcohol); mental disorders or intellectual disabilities; assisted reproductive technology conceptions; multiple gestations; metabolic/reproductive disorders (e.g., polycystic ovary syndrome, hyperlipidemia); significant obstetric history (congenital anomalies, recurrent pregnancy loss); abnormal fetal anomaly scan between 20–24 weeks; major medical comorbidities (malignancy, bariatric surgery, preeclampsia); pre-pregnancy or early-pregnancy hypertension (SBP ≥ 140 mmHg and/or DBP ≥ 90 mmHg); incomplete OGTT or insufficient blood samples; and unavailable data.

**Figure 1:**
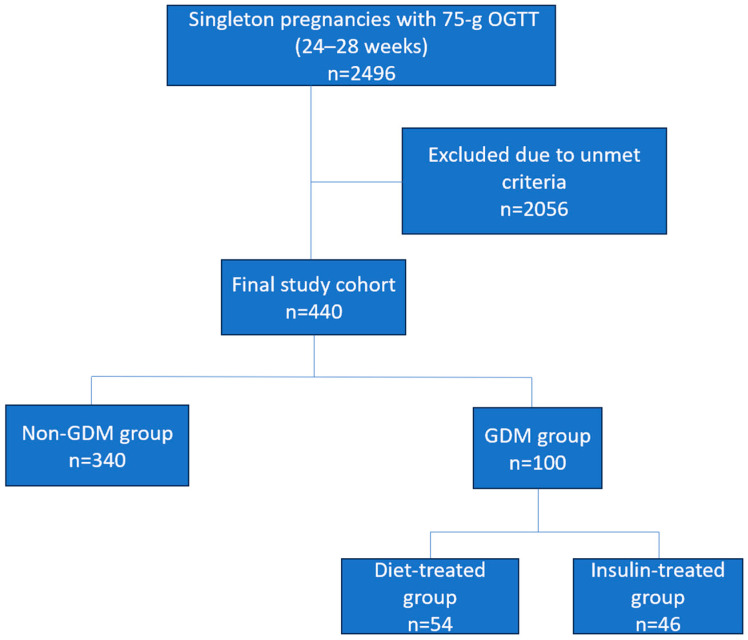
Flow diagram illustrating patient selection and study design.

The patient selection process is summarized in Figure 1.

Overall, all eligible pregnant women who underwent a 75 g OGTT between 24 and 28 gestational weeks were considered for inclusion. After applying the predefined exclusion criteria detailed above, the final analytical cohort was established. Patients diagnosed with GDM were further categorized based on the treatment received: diet therapy only or insulin therapy. Baseline characteristics and established GDM risk factors, including maternal age, body mass index (BMI) at the time of the OGTT, obstetric history, education level, comorbidities, family history of diabetes, smoking status, blood pressure, gestational weight gain, and laboratory measurements (glucose, insulin, lipid profile, and liver and kidney function tests), were recorded for all participants. Data were extracted from electronic medical records by two independent investigators using a prespecified standardized form. Any discrepancies were resolved through consensus or by re-evaluating the original records. This process ensured high consistency in data collection.

All laboratory assessments were performed on fasting blood samples prior to GDM diagnosis or initiation of any treatment. Venous plasma glucose was measured on a Cobas C702 analyzer (Roche Diagnostics GmbH, Mannheim, Germany) at fasting, 60, and 120 min during a 75 g OGTT. GDM was diagnosed using IADPSG criteria [27] defined by at least one abnormal value: fasting ≥ 92 mg/dL (5.1 mmol/L), 1 h ≥ 180 mg/dL (10.0 mmol/L), or 2 h ≥ 153 mg/dL (8.5 mmol/L). Fasting lipid parameters, including TC, triglycerides, and HDL-C, were measured on the same platform, and LDL-C was calculated using the Friedewald formula (LDL = TC − HDL − [TG/5], mg/dL) [28]. Serum insulin was quantified by electrochemiluminescence immunoassay. Insulin resistance was assessed using the Homeostasis Model Assessment (HOMA) index, derived from fasting glucose and insulin levels. The triglyceride-glucose (TyG) index was calculated as ln[fasting triglycerides (mg/dL) × fasting glucose (mg/dL)/2] [1]. Laboratory results and covariates were analyzed between the GDM and non-GDM groups, and a subgroup analysis was conducted to compare GDM cases requiring insulin therapy with those managed with diet therapy.

### Statistical Analysis

All statistical analyses were performed using IBM SPSS Statistics, Version 31. Demographic, obstetric, and laboratory parameters were analyzed as continuous numerical variables and were summarized by mean ± standard deviation (SD) or median and IQR depending on variable distribution. The distribution of each variable was assessed for normality. Based on this assessment, group comparisons were made using the Independent Samples *t*-test for normally distributed data or the Mann-Whitney U test for non-normally distributed data. Categorical variables were summarized as counts and percentages (*n*, %), and associations were evaluated using Pearson’s chi-square test. Analyses were based on complete case data, and no imputation was performed for missing values.

Variables that achieved statistical significance (*p* < 0.05) in these univariate analyses, along with clinically relevant covariates established in the literature, were entered into a multivariable logistic regression model to identify independent predictors of GDM. The model was constructed using the forward likelihood ratio method (*p* < 0.05 for retention). The linearity of continuous variables was assessed using fractional polynomials, and the final model’s goodness-of-fit was confirmed with the Hosmer-Lemeshow test. Multicollinearity was absent, ensuring a model that balanced the risks of overfitting and residual confounding.

The diagnosis of GDM was established using the 75 g OGTT as the reference standard. Receiver operating characteristic (ROC) curve analysis was employed to assess and compare the diagnostic performance of the TyG index, LDL/HDL ratio, TC/HDL ratio, and TG/HDL ratio against this gold standard. The area under the curve (AUC) was calculated for each technique, with 95% confidence intervals. For each parameter, diagnostic performance metrics were calculated at the optimal threshold: sensitivity, specificity, positive predictive value (PPV), negative predictive value (NPV) with 95% confidence intervals. The value of *p* < 0.05 was considered statistically significant.

## 3. Results

A total of 2496 pregnant women with singleton pregnancies who underwent a 75 g OGTT between 24 and 28 gestational weeks were initially identified from the institutional database. Following the application of predefined exclusion criteria, 2056 women were excluded, leaving a final analytical cohort of 440 eligible participants (Figure 1). The overall prevalence of GDM was quantified at 22.7% per IADPSG criteria. Among women diagnosed with GDM, 64% had one glucose value above the diagnostic threshold, 29% had two values above the threshold, and 7% had all three values above the threshold. Of the patients diagnosed with GDM, 46% were administered insulin therapy. A detailed comparison of baseline demographic and clinical characteristics between patients with and without GDM is presented in Table 1.

A comparative analysis of baseline characteristics revealed significant differences between pregnant women with and without GDM. Women diagnosed with GDM were, on average, older (median age: 29.5 vs. 28.0 years; *p* = 0.037) and exhibited a higher BMI at the time of OGTT (27.1 vs. 24.1 kg/m^2^; *p* = 0.007). Furthermore, a family history of diabetes was significantly more prevalent in the GDM cohort (42.0% vs. 28.2%; *p* = 0.009), as was a history of smoking (17.0% vs. 4.7%; *p* = 0.001). Metabolic parameters were also markedly elevated in the GDM group, including fasting insulin (12.2 vs. 8.8 IU/mL; *p* = 0.001), HOMA-IR (2.8 vs. 1.7; *p* = 0.001), fasting triglycerides (213.98 vs. 183.20 mg/dL; *p* = 0.001), and the TyG index (9.1 vs. 8.9; *p* = 0.001). No statistically significant differences were observed for the remaining parameters analyzed.

Variables with *p* < 0.05 in univariate analysis were considered for inclusion in the multivariate model. The following variables met this threshold: age, BMI at OGTT, family history of diabetes, smoking, fasting insulin, HOMA-IR, fasting triglycerides and TyG index. Variable selection for the final multivariable logistic regression model was performed using the forward likelihood ratio method. The final model demonstrated adequate fit, with a Hosmer-Lemes-how test *p* value of 0.520, indicating no significant deviation between predicted and observed outcomes. In the multivariate logistic regression analysis, family history of diabetes was significantly associated with increased odds of GDM (OR = 2.18, 95% CI: 1.15–4.13, *p* = 0.017). Each 1 mg/dL increase in fasting triglycerides was associated with slightly higher odds of GDM (OR = 1.008, 95% CI: 1.003–1.013, *p* = 0.002). Furthermore, each 1-unit increase in the TyG index was associated with a marked increase in the odds of developing GDM (OR = 12.29, 95% CI: 4.26–35.43, *p* < 0.001) (Table 2).

The primary hypothesis that the TyG index would predict GDM was confirmed through multivariable analysis, whereas other serum lipid ratios were not significant predictors. Furthermore, the TyG index was significantly higher in women requiring insulin therapy (*n* = 46) (9.18 ± 0.44) compared to those managed with diet alone (*n* = 54) (8.86 ± 0.35) (*p* < 0.001). Logistic regression analysis revealed that each 1-unit increase in the TyG index was associated with approximately 13 times the odds of requiring insulin (OR = 13.1; 95% CI: 3.31–51.82). However, the wide confidence interval around this estimate indicates that the exact strength of this association should be interpreted with caution. Nonetheless, the findings suggest the TyG index has potential clinical utility in identifying high-risk patients. Furthermore, in the overall cohort, the TyG index demonstrated a significant, moderate positive correlation with both insulin levels (r = 0.39, *p* < 0.001) and HOMA-IR (r = 0.44, *p* < 0.001). In subgroup analyses, this correlation was weaker but remained statistically significant in women without GDM (TyG-insulin: r = 0.30, *p* < 0.001; TyG-HOMA-IR: r = 0.32, *p* < 0.001). By contrast, among women diagnosed with GDM, stronger correlations were observed, with a moderate positive correlation between TyG and insulin (r = 0.40, *p* = 0.003) and a strong positive correlation between TyG and HOMA-IR (r = 0.49, *p* < 0.001). These findings, particularly the higher correlation coefficients observed in women with GDM, support the notion that the TyG index may serve as a practical biomarker reflecting insulin resistance during pregnancy.

**Figure 2:**
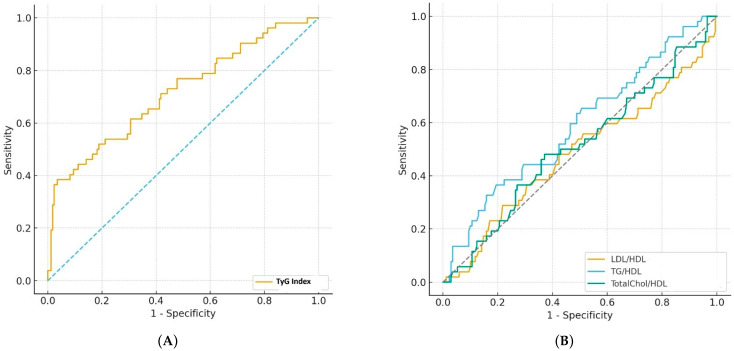
(**A**) ROC curve illustrating the diagnostic performance of the TyG index compared with the reference standard, 75 g OGTT, for the detection of GDM. (**B**) ROC curves illustrating the diagnostic performance of the LDL/HDL, TG/HDL AND TC/HDL ratios compared with the reference standard, 75 g OGTT, for the detection of GDM (Dotted lines indicate the chance diagonal).

The predictive performance of the TyG index and conventional lipid ratios (LDL/HDL, TG/HDL, and TC/HDL) for GDM was assessed using ROC curve analysis. The TyG index demonstrated moderate discriminative ability for overall GDM, with an AUC of 0.716 (95% CI: 0.629–0.798, *p* < 0.001) and an optimal cut-off value of 9.35, yielding a sensitivity of 38.5%, a specificity of 96.5%, and a negative predictive value of 83.7%. Among conventional lipid ratios, TG/HDL exhibited the highest predictive value (AUC:0.587), whereas LDL/HDL and TC/HDL ratios yielded AUC values around 0.50, indicating limited discriminatory capacity (Table 3, Figure 2).

In the subgroup of women diagnosed with GDM, an analysis of diagnostic performance of TyG index was conducted in subjects who did not meet the fasting glucose criterion but met the post-load criterion. The TyG index demonstrated limited discriminative ability overall, with an AUC of 0.639 (95% CI: 0.520–0.748), an optimal cut-off of 9.35, sensitivity of 32.4%, specificity of 96.4%, and a negative predictive value of 87.6%. When analyzed separately, the index showed an AUC of 0.533 (cut-off = 8.55; sensitivity = 92.9%, specificity = 20.6%) for the 1 h (≥180 mg/dL) group and 0.508 (cut-off = 9.39; sensitivity = 33.3%, specificity = 97.6%) for the 2 h (≥153 mg/dL) group.

**Figure 3:**
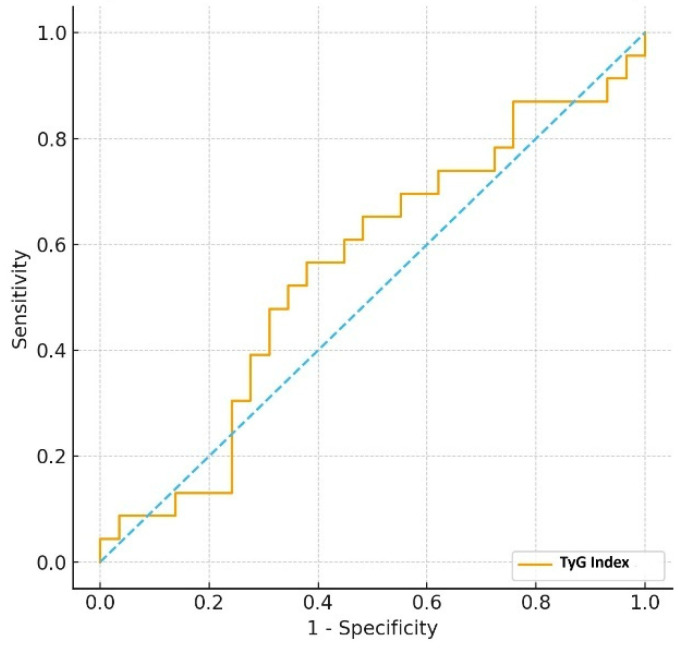
ROC curve of TyG index for distinguishing diet- vs. insulin-treated GDM (Dotted line indicates the chance diagonal).

Furthermore, the ability of the TyG index to differentiate between those managed with diet (*n* = 54) and those requiring insulin therapy (*n* = 46) was assessed. The TyG index demonstrated poor discriminative capacity, with an AUC of 0.553. The optimal cut-off value was determined as 9.16, providing a sensitivity of 56.5% and a specificity of 62.1%, and the negative predictive value was 64.3% (Figure 3).

## 4. Discussion

In this cohort of singleton pregnancies at 24–28 weeks, GDM prevalence was 22.7%, exceeding rates reported in similar populations [18,19,26,29,30,31]. In the multivariate analysis, both the TyG index and a family history of diabetes emerged as independent predictors of GDM. While a family history of diabetes was associated with a more than two-fold increase in odds, the TyG index demonstrated a statistically powerful association, with each one-unit increase corresponding to markedly elevated odds of GDM. However, we acknowledge that the clinical interpretation of this odds ratio requires caution given the actual narrow range of TyG values observed and the small absolute difference (0.2) in medians between groups. The observed statistical significance is primarily driven by the relatively large sample size rather than by a biologically and clinically meaningful difference. Consequently, while the TyG index showed moderate discriminative ability overall (AUC = 0.716), its profile was characterized by relatively low sensitivity but substantially higher specificity. This combination suggests its utility is not in identifying all positive cases but rather in providing meaningful value for ruling out GDM, effectively serving as a potential ‘rule-out’ tool in clinical practice. Conventional lipid ratios performed poorly in GDM prediction.

Our finding of a 22.7% GDM rate can be explained by our center’s practice of universal screening with a 75 g OGTT during weeks 24–28, applied to all pregnant women regardless of their risk profile. The universal screening strategy identifies diabetic cases among all pregnant women, even those lacking standard risk profiles. This, combined with the heightened sensitivity of the IADPSG diagnostic standards, likely resulted in an increased detection rate. Several studies have documented GDM prevalence rates reaching up to 50%, particularly in populations undergoing universal screening with IADPSG criteria [17,32]. The significant variation in reported prevalence can also be attributed to heterogeneity in ethnic composition, diagnostic standards, screening methodologies, and the characteristics of the study populations [33].

There is growing evidence that the TyG index is a valuable marker not only for identifying cardiometabolic, hepatic, and nephrological diseases but also for detecting GDM in pregnant women, as it reflects the degree of insulin resistance [16,18,30,34,35,36,37,38]. While cardiometabolic studies generally report TyG cut-off values of 8.5–8.8 with varying sensitivity and specificity [35,39,40], no universally accepted threshold exists for pregnant populations. In our study, the optimal cut-off for GDM was higher than previously reported. In a nationwide population-based cohort, Kim et al. identified a pre-pregnancy TyG index of 8.15 as a predictor of GDM in the second trimester [18]. Compared with their findings, our cohort showed lower sensitivity (38.5% vs. 47.0%) but markedly higher specificity (96.5% vs. 68.2%), indicating stronger utility for ruling out negative cases in midpregnancy. For insulin requiring GDM, the optimal cut-off in our cohort was also higher (9.16 vs. 8.26), with similar sensitivity (56.5% vs. 57.9%) but lower specificity (62.1% vs. 68.2%), suggesting slightly weaker predictive performance for this subgroup.

These discrepancies may reflect heterogeneous pathophysiological mechanisms of GDM [4,41,42,43,44], differences in study population characteristics and size, or the gestational age at assessment. Notably, TyG assessment in the second trimester captures the profound metabolic and physiological changes that occur as pregnancy progresses, enhancing its ability to detect insulin resistance [18,19]. Early-pregnancy studies have shown smaller risk increases per unit TyG [1]. Kim et al. reported that a one-unit increase in the TyG index was associated with an odds ratio of 1.86 for GDM and 3.93 for insulin-requiring GDM [18], while Pazhohan et al. found a 4.9-fold increased risk independent of other factors [30]. In contrast, our second-trimester cohort demonstrated substantially greater associations, suggesting more reliable prediction at this stage. Consistent with our findings, Mo et al. also found that the TyG index was associated with markedly higher odds of GDM (OR = 12.92) in their first-trimester study. [45]. Furthermore, a recent meta-analysis supported our findings, demonstrating that the TyG index is a promising biomarker for predicting GDM, particularly in the second trimester where it showed low heterogeneity, while no such association was observed in the first trimester [46]. The TyG index’s predictive power increases in the second trimester because the physiological changes in that period, progressive insulin resistance and increased lipolysis, mirror the pathophysiology of GDM itself. Since these changes are minimal in the first trimester, the index’s ability to identify at-risk women is limited early on. However, a limitation of second trimester assessment is the relatively late GDM diagnosis, which narrows the window for preventive or therapeutic interventions aimed at reducing maternal and fetal morbidity and mortality; nevertheless, since most pregnant women do not attend follow-up visits until the second trimester, conducting these analyses at this stage may still provide valuable insights.

Both our study and Sánchez-García et al. assessed the TyG index during the second trimester, supporting the notion that this period is optimal for capturing pregnancy-induced insulin resistance [17]. However, notable differences in diagnostic performance were observed. In Sánchez-García et al. study, the TyG cut-off was 4.68, yielding high sensitivity (89%) but relatively low specificity (50%), indicating strong ability to identify positive GDM cases but limited capacity to rule out negatives. In contrast, our cohort demonstrated a cut-off with markedly lower sensitivity but substantially higher specificity, reflecting better performance in excluding non-GDM cases but reduced ability to detect positive cases. Unlike the study by Sanchez et al., our investigation included a subgroup analysis of women diagnosed with GDM based solely on elevated post-load glucose criteria. The TyG index demonstrated limited discriminative ability in this cohort, exhibiting poor performance in both 1 h and 2 h post-load subgroups and failing to effectively distinguish between patients managed with diet alone and those requiring insulin therapy. We propose that this limited utility stems from several factors. Primarily, the TyG index is inherently reliant on fasting glucose, a parameter that remains normal in this specific phenotype, which explains its inability to capture the significant insulin resistance often present. Furthermore, the index does not account for critical pathophysiological components of GDM, such as postprandial glucose excursions driven by β-cell dysfunction rather than pure insulin resistance. The clinical heterogeneity of GDM, influenced by factors like maternal weight, gestational age, and family history, likely further diminishes the index’s sensitivity. These findings suggest that, while the TyG index is a robust marker for identifying insulin resistance and GDM diagnosis, its utility diminishes after diagnosis.

The progression to requiring insulin therapy is determined by a complex interplay of factors beyond insulin resistance, including β-cell reserve and secretory capacity. Therefore, the TyG index alone is insufficient for predicting therapeutic needs or identifying GDM subtypes, underscoring the necessity for more comprehensive, population-specific, and pathophysiology-oriented tools to guide clinical management. Its sensitivity and specificity in distinguishing women who will require insulin therapy may be significantly improved through its integration with other surrogate markers of insulin resistance. Future investigations should aim to establish different models that integrate the TyG index with other readily available clinical indicators. Such an approach may enhance the precision of GDM risk stratification and support individualized management strategies, rather than relying on a single biomarker. Indeed, studies demonstrating improved diagnostic performance have been reported [38,47].

While conventional lipid ratios, including TG/HDL, LDL/HDL, and TC/HDL, are commonly used as proxies for insulin resistance and cardiometabolic risk, their effectiveness in predicting GDM remains inconclusive. Although numerous studies have reported a positive association between these ratios and GDM risk, their prognostic value is generally modest and less robust than that of the TyG index [11,12,13,23,24,26]. This limitation may stem from pregnancy-specific metabolic changes, such as physiologic hyperlipidemia, which can obscure the utility of conventional lipid measures. Moreover, the absence of validated gestational cut-off values restricts their clinical applicability. Collectively, these findings suggest that while conventional lipid ratios provide limited insight for predicting GDM, the TyG index demonstrates superior discriminative capacity. Consistent with our study, TG/HDL has been identified in previous research as the most prominent conventional lipid ratio for predicting GDM [11,26].

The retrospective design of this study imposes several inherent limitations. First, potential selection bias may have influenced the composition of the study cohort, as inclusion depended on predefined criteria and available medical records. Second, variations in sample collection, handling, and testing procedures inherent to routine clinical practice, along with the absence of longitudinal laboratory quality control data, may have introduced inconsistencies despite standardized protocols. Third, due to the observational nature of a retrospective study, causal relationships cannot be firmly established. Fourth, although participants with certain health conditions were excluded, exclusion was based solely on patient-reported medical histories. Detailed review of hospital records for other conditions potentially affecting blood glucose, lipid levels, or insulin secretion was not performed, making it challenging to fully ensure the absence of underlying diseases that could influence the study outcomes. Fifth, the relatively small sample size may have limited the statistical power to detect modest associations. Sixth, this study was conducted at a single center in Van, Türkiye, a relatively less developed region, which may limit the generalizability of the findings to more developed settings. Future research should include populations from economically diverse regions, with larger cohorts, wider age ranges, and subgroup analyses by relevant factors, to enhance the external validity of the results. Moreover, our analysis did not account for potential confounders such as prepregnancy lipid profile, genetic variations, factors involved in the pathogenesis of GDM (e.g., adiponectin, leptin, and adipocyte fatty acid-binding protein, etc.) previous obstetric complications, socioeconomic status, dietary intake, physical activity, sleep quality, and mental health. These limitations should be considered when interpreting the results and generalizing findings to broader populations.

## 5. Conclusions

The TyG index is a strong independent predictor of GDM in the second trimester. While its low sensitivity limits its utility as a universal screening tool, its high specificity makes it an excellent “rule-out” test. A low TyG value can reliably identify women at very low risk for GDM, enabling more efficient allocation of diagnostic resources. Furthermore, the TyG index demonstrated superior predictive performance compared to conventional lipid ratios. Future studies should focus on developing integrated models that combine the TyG index with other clinical factors to enhance predictive accuracy and clinical utility.

## Figures and Tables

**Table 1:** Baseline anthropometric and biochemical characteristics of the study cohort.

	No GDM *(n* = 340)	GDM (*n* = 100)	*p* Value
Age, years, median [IQR]	28 [9.0]	29.5 [7.0]	**0.037**
BMI at OGTT, kg/m^2^, median [IQR]	24.1 [6.1]	27.1 [5.9]	**0.007**
Gravidity\Parity, median [IQR]	2 [2]/1 [2]	2 [2]/1 [2]	0.390
Education level, *n* (%)			0.098
<High school	195 (57.4)	48 (48)	
≥High school	145 (42.6)	52 (52)	
Comorbidity, *n* (%)			0.834
No	300 (88.2)	89 (89)	
Yes	40 (11.8)	11 (11)	
Family history of diabetes, *n* (%)			**0.009**
No	244 (71.8)	58 (58)	
Yes	96 (28.2)	42 (42)	
Smoking, *n* (%)			**0.001**
No	324 (95.3)	83 (83)	
Yes	16 (4.7)	17 (17)	
Systolic blood pressure, mm Hg, mean ± SD	103.82 ± 9.21	102.11 ± 9.76	0.324
Diastolic blood pressure, mm Hg, mean ± SD	66.20 ± 7.96	64.71 ± 8.48	0.303
Gestational weight gain at OGTT, kg, median [IQR]	7 [7]	7 [6]	0.788
Gestational age at OGTT, week, median [IQR]	26.1 [2.6]	26.1 [3.8]	0.651
Fasting plasma glucose, mg/dL, mean ± SD	78.34 ± 5.94	91.88 ± 14.45	**0.001**
1 h plasma glucose, mg/dL, mean ± SD	126.05 ± 25.05	172.00 ± 31.36	**0.001**
2 h plasma glucose, mg/dL, mean ± SD	108.00 ± 18.26	135.71 ± 31.75	**0.001**
Fasting insulin, lU/mL, median [IQR]	8.8 [5.7]	12.2 [7.9]	**0.001**
HOMA-IR, median [IQR]	1.7 [1.2]	2.8 [2.1]	**0.001**
Fasting triglycerides, mg/dL, mean ± SD	183.20 ± 57.93	213.98 ± 66.56	**0.001**
Fasting HDL, mg/dL, median [IQR]	63 [22.3]	62 [20.8]	0.623
Fasting LDL, mg/dL, mean ± SD	121.42 ± 29.35	118.73 ± 37.12	0.587
Fasting cholesterol, mg/dL, mean ± SD	222.59 ± 35.61	225.15 ± 45.45	0.672
TyG index, median [IQR]	8.9 [0.5]	9.1 [0.5]	**0.001**
LDL/HDL ratio, median [IQR]	1.9 [0.8]	1.9 [1.0]	0.544
Cholesterol/HDL ratio, median [IQR]	3.5 [1]	3.6 [1.2]	0.895
Triglyceride/HDL ratio, median [IQR]	2.8 [1.7]	3.2 [2.4]	0.053
AST (mmol), median [IQR]	14.3 [5.8]	14 [5.8]	0.408
ALT (mmol), median [IQR]	13 [8.1]	13 [6.1]	0.859
BUN (mmol/L), median [IQR]	14.2 [4.6]	14.9 [6.2]	0.616
Serum creatinine, median [IQR]	0.5 [0.1]	0.5 [0.1]	0.335

Note: AST: Aspartate Aminotransferase; ALT: Alanine Aminotransferase; BMI: Body mass index; GDM: Gestational diabetes mellitus; HOMA-IR: Homeostatic Model Assessment of Insulin Resistance; IQR: Interquartile Range; OGTT: Oral glucose tolerant test; HDL: High density lipoprotein; LDL: Low density lipoprotein; SD: Standard deviation; TyG index: Triglyceride and glucose index.

**Table 2:** Logistic regression analysis of factors for predicting GDM.

Variable	Coefficient	SE	Wald	df	*p*	OR	95% Confidence Interval (OR)	
							Lower	Upper
Family history of diabetes *	0.779	0.326	5.698	1	**0.017**	2.179	1.149	4.129
Fasting triglycerides	0.008	0.003	9.516	1	**0.002**	1.008	1.003	1.013
TyG index	2.509	0.540	21.579	1	**<0.001**	12.291	4.264	35.425
Constant	−23.685	4.880	23.558	1	**<0.001**	0.001		
* Reference category: No					CCR = 82.6% CCR = 82.6% χ^2^_(5)_ = 6.169; *p* = 0.520			

Note: SE: standard error; df—degree of freedom; OR—odds ratio; Explanations: The Hosmer-Lemeshow goodness-of-fit test yielded a *p* value of 0.520, indicating no significant deviation between the model-predicted probabilities and observed outcomes, thereby supporting adequate calibration of the logistic regression model. A value close to 1 indicates a good fit, while a value significantly different from 1 suggests a poor fit.

**Table 3:** Diagnostic performance of the TyG index and conventional lipid ratios for the detection of GDM.

Measure	TyG Index > 9.34 *n* = 440	LDL/HDL > 2.39 *n* = 440	TG/HDL > 4.04 *n* = 440	TC/HDL > 3.73 *n* = 440
True positive	35	30	38	50
False positive	12	73	66	125
True negative	328	264	270	212
False negative	65	73	66	53
Sensitivity [95% CI]	35.0 [26.4–44.7]	29.1 [20.3–37.9]	36.5 [27.2–45.8]	48.5 [38.9–58.1]
Specificity [95% CI]	96.5 [93.9–98.0]	78.3 [73.9–82.7]	80.4 [76.2–84.6]	62.9 [57.8–68.1]
Positive predictive value, %	74.5	28.8	36.5	28.4
Negative predictive value, %	83.5	78.2	80.4	79.9
Positive likelihood ratio [95% CI]	9.9 [5.4–18.4]	1.34 [0.93–1.93]	1.86 [1.33–2.59]	1.31 [1.03–1.67]
Negative likelihood ratio [95% CI]	0.7 [0.6–0.8]	0.91 [0.79–1.04]	0.79 [0.68–0.92]	0.82 [0.67–1.01]
AUC [95% CI]	0.710 [0.627–0.793]	0.483 [0.383–0.579]	0.587 [0.496–0.672]	0.509 [0.417–0.599]
Overall diagnostic accuracy, %	82.5	66.8	70.0	59.5
Diagnostic odds ratio [95% CI]	14.7 [7.3–29.9]	1.49 [0.91–2.45]	2.36 [1.46–3.82]	1.60 [1.03–2.50]

Note: AUC: Area under curve; CI: confidence interval; HDL: High-Density Lipoprotein; LDL: Low-Density Lipoprotein; TG: Triglyceride; TC: Total Cholesterol; TyG: triglyceride and glucose index.

## Data Availability

The authors confirm that they have adhered to their institution’s protocols regarding the publication of patient data in this study. All data generated or analyzed during this study are presented within this article. The data supporting the findings of this study are available upon request from the corresponding author, but they are not publicly accessible due to privacy and ethical constraints.

## Abbreviations

The following abbreviations are used in this manuscript:

ALT	Alanine Aminotransferase
AST	Aspartate Aminotransferase
AUC	Area Under Curve
BMI	Body Mass Index
CI	Confidence Intervals
DBP	Diastolic Blood Pressure
DF	Degree Of Freedom
HOMA-IR	Homeostatic Model Assessment Of Insulin Resistance
GDM	Gestational Diabetes Mellitus
IADPSG	International Association Of The Diabetes And Pregnancy Study Groups
IQR	Interquartile Range
IUI	Intrauterine Insemination
IVF	In Vitro Fertilization
LDL	Low Density Lipoprotein
HDL	High Density Lipoprotein
NPV	Negative Predictive Value
OGTT	Oral Glucosetolerance Test
OR	Odds Ratios
PPV	Positive Predictive Value
PCOS	Polycystic Ovary Syndrome
ROC	Receiver Operating Characteristic
SBP	Systolic Blood Pressure
SD	Standard Deviation
SE	Standard Error
TC	Total Cholesterol
TG	Triglyceride
TyG	Triglyceride-Glucose
VLDL	Very Low Density Lipoprotein
